# The effects of different sample storage conditions on faecal corticosterone metabolite measurements in northern bobwhite (*Colinus virginianus*)

**DOI:** 10.1093/conphys/coaf051

**Published:** 2025-07-29

**Authors:** Hannah N Suber, Jeremiah Leach, Ashley Kaskocsak, Henry Valencia, Sarah Colette, Ronald J Kendall

**Affiliations:** Wildlife Toxicology Laboratory, Texas Tech University, 1234 Davis Dr., Lubbock, TX, 79410, USA; Wildlife Toxicology Laboratory, Texas Tech University, 1234 Davis Dr., Lubbock, TX, 79410, USA; Wildlife Toxicology Laboratory, Texas Tech University, 1234 Davis Dr., Lubbock, TX, 79410, USA; Wildlife Toxicology Laboratory, Texas Tech University, 1234 Davis Dr., Lubbock, TX, 79410, USA; Wildlife Toxicology Laboratory, Texas Tech University, 1234 Davis Dr., Lubbock, TX, 79410, USA; Wildlife Toxicology Laboratory, Texas Tech University, 1234 Davis Dr., Lubbock, TX, 79410, USA

**Keywords:** Animal physiology*, Colinus virginianus*corticosterone, environmental conditions, enzyme immunoassay, faecal corticosterone metabolites, faecal glucocorticoids, northern bobwhite, stress

## Abstract

The northern bobwhite (*Colinus virginianus*) is an economically and ecologically vital gamebird in North America experiencing vast population declines. With the recent validation of an enzyme immunoassay to detect corticosterone metabolites in faeces, there are many opportunities for its scientific application. Corticosterone, a key avian stress-related hormone, has many beneficial functions that support a quail’s immune response, primarily by suppressing inflammation, allowing cells to function more efficiently. However, chronic levels of elevated corticosterone in Aves have been shown to cause metabolic disruption and suppressed reproduction and growth. Determining root causes of chronically elevated corticosterone levels is vital for bobwhite conservation efforts. Proposed research investigating causes of bobwhite stress includes examining the effects of pesticides, climate, disease and management strategies. However, the various methodologies exploring these relationships may result in different ways the faeces are stored and processed, especially in studies on wild bobwhite. These differences may impact research outcomes leading to incorrect conclusions. This study was conducted to determine if enzyme immunoassay results from faecal samples frozen or left in the environment before extraction of faecal corticosterone metabolites differ from those where extraction is immediate. Faeces treatments affected the corticosterone metabolite measurements differently depending on whether the faeces were from males or females, so the effects of treatments were analysed within each sex. No significant difference was found in female faecal corticosterone metabolite concentrations between the frozen and environmentally exposed faeces (*P* = 0.853); however, concentrations in the immediately extracted faecal corticosterone metabolites were significantly lower (*P* < 0.001). Male bobwhite faecal samples that were immediately frozen had significantly lower faecal corticosterone metabolite concentrations compared to environmentally exposed male samples and frozen female samples (*P* = 0.039). These results indicate that faecal corticosterone metabolite concentrations are comparable between environmentally exposed samples from both sexes and frozen samples from females.

## Introduction

The northern bobwhite (*Colinus virginianus*) is an ecologically, economically and culturally valuable gamebird that has experienced notable declines over the last few decades (Johnson *et al.,* 2012; Hernández *et al.,* 2013; Crosby *et al.,* 2015; Sauer *et al.,* 2017). Due to the bobwhite population declines, the number of hunters pursuing these birds have also decreased. This has resulted in millions of dollars being lost or diverted from the rural economies, small towns and conservation funds that depend on the annual influx of hunters purchasing hunting licences, equipment, food and lodging (Burger *et al.,* 1999; Johnson *et al.,* 2012). Furthermore, if landowners do not notice bobwhite population increases after investing resources in bobwhite habitat management, these management efforts will likely decrease. As an umbrella species, bobwhite requires a diverse and structurally complex habitat that also supports numerous other species within the same ecological community (Crosby *et al.,* 2015). Reductions in bobwhite-focused habitat management are expected to result in decreased habitat quality for many other species, negatively affecting local biodiversity. Therefore, maintaining and enhancing northern bobwhite populations is critical not only for the species itself but also for the conservation of broader ecological communities.

Measuring adrenocortical activity in wildlife through assessing glucocorticoid (GC) production is a useful tool way to assess the effects of various factors on the fitness of a variety of taxa. GCs are biomarkers of energy mobilization and often increase due to physiological responses to stress (Noushad *et al.,* 2021). For example, birds and mammals in disturbed forests had higher GC levels than those in undisturbed forests, with species defined as ‘Threatened’ under the IUCN Red List having a much larger response than species of ‘Least Concern’ (Messina *et al.,* 2018). The differences in the degree of stress response could help identify which species are most at risk for a significant population decline due to habitat disturbance. Jankowski *et al*. (2014) found a positive correlation between cattle pat count and sage-grouse (*Centrocercus urophasianus*) stress hormone metabolites in the western USA, inferring that heavy grazing could be contributing to the sage-grouse population declines. Creel *et al.* (2009) found that elk faecal GC concentrations were not related to predator–prey ratios, inferring that, in the system investigated, exposure to predators does not cause chronic stress in elk. Baos *et al.* (2006) determined that sublethal lead exposures were positively correlated with increased adrenocortical stress responses in nestling white storks (*Ciconia ciconia*), highlighting another way this heavy metal may be impacting wildlife species. These and many more similar studies help guide the implementation of management strategies in various circumstances.

The impacts of stressors on organisms, as described in the aforementioned studies, are typically assessed through the measurement of allostatic load, which reflects the cumulative effects of both acute stressors, such as predator evasion, and chronic stress on overall health (Seeley *et al.,* 2022; Guidi *et al.,* 2020; Beckie, 2012). Glucocorticoids are primarily produced to allow organisms to maintain energy homeostasis, often in spite of stressors. Typical adrenocortical activity in response to short-term stress-inducing events benefits individuals, as GC stress hormones are released in response to acute stressors to increase fitness. In these cases, some functions are altered to allow an individual to quickly counteract the impact of a stressor (e.g. increased heart rate, increased respiration, muscle tension, etc.), and normal physiological activities are resumed after the stressor has passed (Ladewig, 2000). However, these same stress responses can be harmful to individuals in the presence of chronic stressors. Long-term elevated stress levels often lead to decreased fitness. The constant production of stress hormones can lead to increased energy demands, resulting in higher food demands that individuals can no longer meet beyond a certain threshold. Furthermore, chronically elevated stress hormone levels can suppress immune responses, disrupt reproductive behaviour and the reproductive hormone axis, decrease digestive efficiency and more (Seeley *et al.,* 2022; Béziers *et al.,* 2020; Blas, 2015; Nelson *et al.,* 2015; Beckie, 2012). Determining the causes of chronic stress and an organism’s allostatic load in vulnerable wildlife populations is a vital research initiative.

It is worth stating that conclusions should not be drawn from detected GC levels, but rather, this data should be used to indicate that further investigation into the factors correlated with elevated GCs is warranted. Elevated GC levels result from a complex interplay of environmental and physiological influences and may not necessarily indicate reduced fitness, as stress responses are often within an organism’s allostatic capability (Dantzer *et al.,* 2014; Romero *et al.,* 2015). Without a deeper understanding of these contributing elements, any assumptions made based on GC measurements alone could be misleading. Therefore, identifying elevated stress-associated hormone levels should serve as a starting point for more comprehensive assessments of the impacts of factors, allowing for a more accurate interpretation of organism well-being within a broader context.

Traditional procedures to ascertain animal GC levels, like blood collection, can be invasive and cause undue stress on the subject. Furthermore, blood GCs are the active form of the hormones, representing the current amount being utilized by an organism (Noushad *et al.,* 2021). Spikes in GC levels caused by capture or disturbance can distort the data, impacting study results. Alternative methods, like measuring GC metabolite concentrations in urine or faeces, are often advantageous as they are much less invasive and more feasible to collect. Glucocorticoid metabolite levels represent an average level of circulating GCs over a recent time frame rather than a point sample (Harper and Austad, 2000). Furthermore, there is a delay between the rise in GC levels in an organism and the corresponding increase in metabolites in the faeces, which in Aves is between 2 and 24 h (Legagneux *et al.,* 2011; Palme *et al.,* 2005; Wasser *et al.*, 2000). This gives researchers more time to collect samples before any spikes in the measured GC levels occur. Therefore, measuring GC metabolites in excreta is often preferred in studies investigating GC levels in wildlife populations.

With the recent validation of an enzyme immunoassay (EIA) to detect metabolites of the avian stress hormone corticosterone in faeces (Leach *et al.,* 2024), there are many opportunities for its application toward the goal of restoring bobwhite populations. Proposed research using faecal corticosterone metabolite (FCM) measurements aims to investigate how climate, disease, parasitic infection and management strategies may impact stress in bobwhite quail, and how this stress affects breeding success and survival. These varied goals may lead to differences in how faecal samples, especially from wild bobwhite, are stored and processed. Wild bobwhite are often trapped over multiple days or weeks, and sample processing materials are not always on the site of capture. This means corticosterone metabolites would be extracted immediately from the most recently obtained faecal samples while the earliest samples collected would be frozen for a period before extraction occurs. Additionally, faeces not immediately collected from a bird (e.g. collected from around a nest for a reproduction study) would be exposed to a variety of environmental conditions (UV, bacterial degradation, temperature fluctuations, etc.) that may affect EIA results (Cantalupi *et al.,* 2020; Carbillet *et al.,* 2023).

Proper storage of faecal samples has been shown to be a vital part of conducting GC immunoassays, as different storage techniques have produced varied results (Kahn *et al.,* 2002; Washburn and Millspaugh, 2002; Carbillet *et al.,* 2023). Möstl and Palme (2002) observed bacterial degradation of steroid metabolites within a few hours, while Messmann *et al.* (1999) found that storage at room temperature for no more than 30 days does not significantly affect assay results for GCs. Freezing faecal samples is a common method of halting microbial degradation, but the difference between frozen and immediately processed samples has not been adequately investigated. Thus, it is difficult to hypothesize if the different treatments on the faecal samples will affect the EIA results. The purpose of this study is to determine if EIA results from faecal samples that were frozen or left exposed in the environment before corticosterone extraction differed from samples where corticosterone was immediately extracted to widen the applicability of the assay in field research.

## Materials and Methods

### Animal care and ethics

Eighteen pen-reared adult bobwhite (9 male and 9 female), 13 months old, were used for this study and maintained under Texas Tech University Institutional Animal Care and Use Committee protocol 2022–1178. Birds were housed individually in breeding batteries, provided food (Purina® Crumbles Game Bird Maintenance Feed) and water *ad libitum* and maintained under an 18:6 light:dark cycle.

### Sample collection and treatment

Bobwhite were divided into three groups of six (Groups A, B and C), with three males and three females in each group. Over three consecutive days beginning at 0800 hrs, an aluminium foil sheet was placed under each cage so fresh droppings from the individuals could be collected. All faeces were collected from the foil at 0900 hrs and then again at 1000 hrs, resulting in a 1-h maximum collection window. These times were chosen as previous research demonstrated negligible variation in baseline FCM levels between these 2 h and between days at these times (Leach *et al.,* 2024). Each piece of faecal matter was stored separately, labelled with bird ID, date and time collected, with a target sample size of three faeces/individual during each collection time. On Day 1, the faeces from Group A were dried and FCM were extracted immediately following the methods outlined in Leach *et al.,* 2024. Faeces were placed in 14-l latch boxes with 112 g desiccation patch until dry, which took no more than 24 h. Each piece of faeces was ground into a fine powder via mortar and pestle until homogenized, then placed into 2-ml centrifuge tubes aliquoted into 100- to 150-mg samples. Sixty percent EtOH was added to the sample at a ratio of 1 ml for every 100 mg of faeces. Then samples were vortexed for 30 min and centrifuged at 4°C for 15 min. The supernatant was transferred into a new centrifuge tube and placed back into the latch box with the desiccation packs until all ethanol was evaporated. Samples were reconstituted with 50 μl of 60% EtOH and diluted with 750 μl of the provided assay buffer then quantified using Arbor Assays™ Detect-X© corticosterone EIA kits (CAT 50–227-8401). The plate was analysed using the Byonoy™ Absorbance 96® microplate reader at 450 nm.

**Figure 1:**
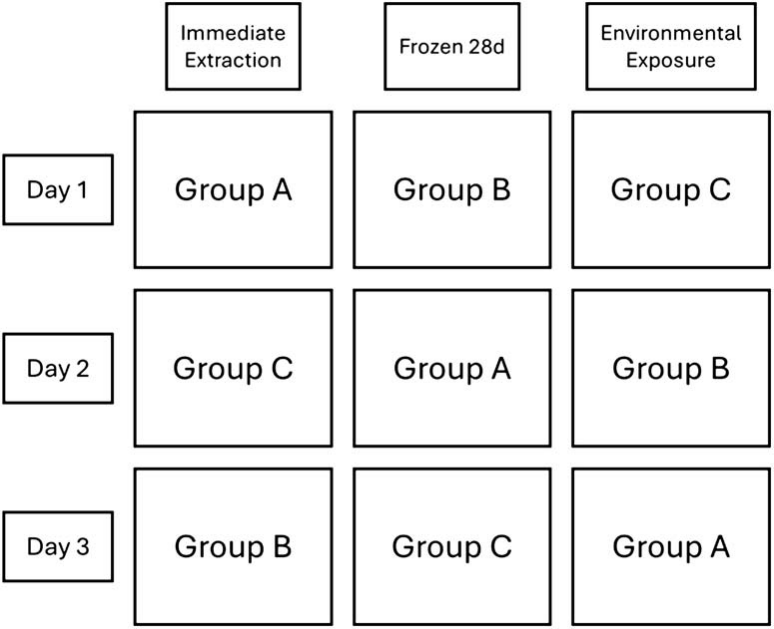
Schematic of the treatment the faeces from each group received each day.

The faeces from Group B were frozen at −20°C for 28 days, then simultaneously thawed and dried, and the FCM extracted. The faeces from Group C were placed on a weigh boat and left outside for 24 h before FCM extraction. The samples left outside were exposed to sunlight as it naturally occurred. Temperature, humidity and cloud cover data were monitored across all 3 days of treatment. On Days 2 and 3, the faeces from each group received a different treatment, so each individual’s faeces received each treatment (Fig. 1). Treatments were rotated to account for individual variation in FCM levels. Multiple groups were used to account for daily variation in FCM levels. The environmental exposure treatment occurred 28–30 May in Lubbock, Texas. The average high across days was 30.2 ± 0.3°C, the average low was 16.9 ± 1.1°C and the average humidity was 63.8 ± 1.1%. All days were partly cloudy (Weather Underground, 2024).

### Analysis

The raw output of the EIA was transformed in accordance with the manufacturer’s recommendation to get the concentration of metabolites in picogram per gram faeces. Faecal corticosterone metabolites from samples collected from the same individual at the designated time points were extracted and analysed separately. The data were then averaged within individual and time point to get an average FCM from each individual at each time point. The final concentration was then transformed using the natural log. Analysis was done using R Studio® version RStudio 2024.04.2 + 764 (RStudio Team, 2024).

Data were analysed using a global mixed-effects generalized linear mixed model with the lmer() function from the lme4 package (v1.1–33.5; Bates *et al.,* 2015). Individual bobwhite and dates the faeces were collected were included as random effects with random intercepts and fixed means. Fixed effects included storage condition, sex, time and all two-way interactions among these factors. Model selection was based on the Akaike Information Criterion corrected for small sample sizes (AICc), using the dredge() function from the MuMIn package (Bartoń, 2024). Competing models were identified as those with ΔAICc < 2 and were further assessed for suitability. This included visual checks of model assumptions using the R plot_model() function and the examination of correlations among fixed effects. Point estimates of the factors were calculated along with confidence estimates using the recommendations found in Gellman (2008). Confidence intervals (CIs) were used to determine if treatment effects were significant and cross-checked with *P*-value. Effects were considered statistically significant from their respective reference condition if their CIs did not include zero and if *P* < 0.05; otherwise, they were considered not significantly different from the reference condition.

## Results

Faecal corticosterone concentrations were measured from a total of 197 faecal samples. After averaging samples within individuals and collection times, 100 distinct FCM estimates were used in the analysis. At least one sample from each individual bobwhite and each treatment (environmental, frozen and immediate extraction) were used in the analysis. Of the 100 estimates, 31 came from faeces stored in environmental conditions, 35 from frozen faeces and 34 from faeces from which the FCMs were extracted immediately. The average FCM estimates of all samples was 7625 pg/g faeces, and the standard deviation was 3553 pg/g faeces. Intra-assay variation was 6.34% and inter-assay variation was 6.47%.

The model using sex, faeces treatment and the interaction between the two variables as predictors for FCM concentration was the top model based on AICc score (ΔAIC = 0). The top six models from the model selection output are displayed in S1. The only competing model included time and the interaction of time and sex as well (ΔAIC = 1.05); however, an examination of fixed effect correlation showed that both those factors were highly correlated with other factors in the top model. Thus, it was determined that treatment of the faeces did affect the detected level of FCM, time collected did not and that the effects of treatment should be analysed within each sex.

**Figure 2:**
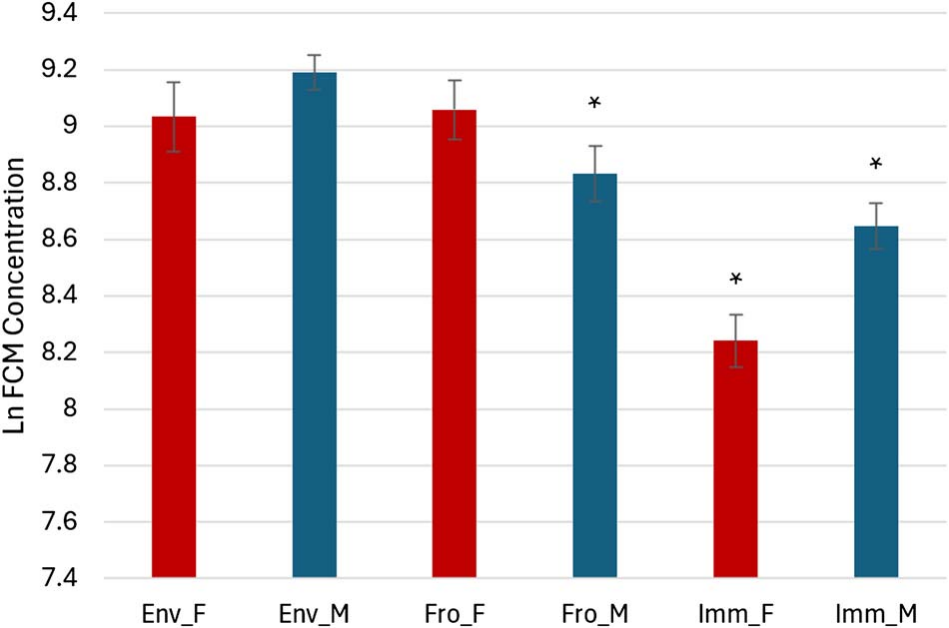
Estimated Ln FCM concentrations using an EIA of male (blue) and female (red) bobwhite when faecal samples are stored under different conditions. Asterisk denotes significance (*P* < 0.05). Env = 24 h under environmental conditions, Fro = immediately frozen, Imm = immediately extracted, M = male and F = female.

**Figure 3:**
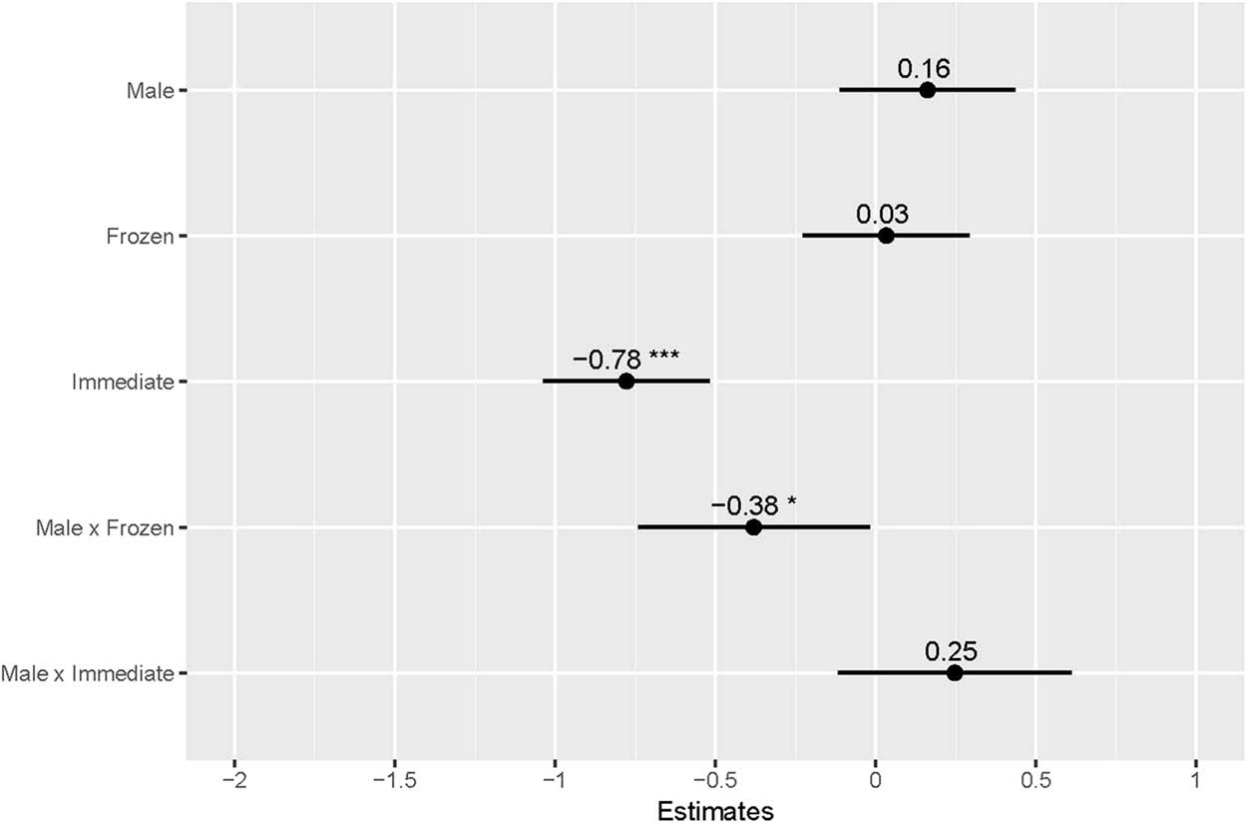
Figure of estimates and confidence intervals of fixed effects of bobwhite faecal samples stored under different conditions. Females and 24-h under environmental conditions were the reference conditions. ‘*’ denotes 0.05 > *P* > 0.01. ‘***’ denotes *P* < 0.001. FCM levels detected in environmentally exposed faeces from both males and females, as well as in frozen faeces from females, were not significantly different. FCM levels were significantly lower in frozen male faeces and in faeces immediately extracted from either sex.

The ln-transformed means of FCM values with 95% CIs for each treatment from each sex are shown in Fig. 2. The point estimates along with their associated CIs for the mixed effects are presented in Table 1 and Fig. 3; the estimates of random effect variation are listed in Table 2. Females and faeces exposed to environmental conditions for 24 h were the reference conditions. The fixed effects explained 39.3% of the data variation; fixed and random effects together explained 46.2% of the variation. FCM concentrations were significantly lower when extracted immediately from female samples compared to freezing or 24-h exposure (*P* < 0.001), with no difference between the latter two (*P* = 0.853). In males, frozen samples had significantly lower FCM levels than environmentally exposed male samples and frozen female samples (*P* = 0.039). This means that FCM concentrations in environmentally exposed faeces from both sexes, and frozen faeces from females, were comparable, while the frozen male samples and immediately extracted samples from either sex showed significantly lower concentrations.

**Table 1:** Table of estimates and confidence intervals of fixed effects of bobwhite faecal samples stored under different conditions. Females and 24-h under environmental conditions were the reference conditions. *n* = 9 males and 9 females.

	***Estimate***	***2.5%***	***97.5%***
**Male**	0.1556	−0.1125	0.4241
**Frozen**	0.0254	−0.2364	0.2860
**Immediate**	−0.7885	−1.0540	−0.5301
**Male × Frozen**	−0.3787	−0.7465	−0.0178
**Male × Immediate**	0.2469	−0.1175	0.6153

**Table 2:** Random effects estimates of the top AICc selected model of FCM concentrations in bobwhite faecal samples exposed to different storage conditions.

***Random effects***	***Estimates***
**σ2**	0.14
**τID**	0.01
**τDate**	0.01
**ICC**	0.11
**NID**	19
**NDate**	3
**Observations**	100
**Marginal R2/Conditional R2**	0.393/0.462

## Discussion

Here, we determined the effects of faeces storage prior to FCM extraction using a previously validated EIA. Measuring FCM is a simple, non-invasive technique for measuring stress-related hormones in wildlife, including bobwhite, an economically and ecologically vital gamebird. However, standardizing sampling and storage methods for biological materials from wild bobwhite, which often occurs in remote areas, can be challenging. There may be limitations in access to equipment like freezers, difficulty maintaining consistent sampling times and difficulty obtaining enough samples from captured birds compared to more passive sampling methods. Therefore, these data will be especially valuable to research involving field collections, providing clarity on which sample types are comparable.

Since storage conditions affect the detected FCM levels in faeces collected from males, methods using male faecal samples to investigate the effects of factors on bobwhite stress-related hormones on bobwhite must be standardized. To be comparable, all male faeces must be collected and stored using the same methods. Although no significant differences were found between males and females in the environmentally exposed faeces, future studies using either frozen or freshly extracted faeces should still account for the potential influence of sex on the results, as it may affect outcomes under different conditions. The lack of difference between the 24-h environmental exposure and the 28-day frozen samples from females broadens the applicability of the EIA studying females. For example, faeces could be collected from an unattended nest for a reproduction study and compared to faeces from a captured hen frozen before extraction. This would allow for a comparison between nesting and non-nesting females without having to capture individuals each time a sample is needed.

The differences between treatments may be due to bacterial degradation and various biochemical processes. Glucocorticoids like corticosterone are metabolized into their respective faecal metabolites, in part by gut microbiota, which would also be found in the faeces (Bokkenheuser and Winter, 1980). The microbial biotransformation of corticosterone into FCMs was likely halted in the immediately extracted faeces. In contrast, the frozen faeces allowed some further microbial biotransformation to occur before faeces were fully frozen and during thawing, and the environmentally exposed samples allowed microbial action to occur the longest. In addition, the final FCM concentrations could also be increased by the release of corticosterone from lipid micelles over time, giving the microbiota more material to metabolize (Jensen *et al.,* 2010). The difference between the males and females may be due to faeces size, as female faeces are often larger leading to increased drying and freezing times. This longer freezing or drying time for female may have allowed for more degradation of corticosterone into the FCM.

These results may only apply to warm, semi-arid environments since an environmental exposure under humid conditions did not occur. Changes in detected FCM levels would be consistent with results of other research. For example, simulated rainfall has resulted in significantly higher concentrations of FCM in free-ranging white-tailed deer (Washburn and Millspaugh, 2002), so the possibility of similar effects occurring in bobwhite faeces should not be discounted. Artificially inflated FCM levels due to untested effects on EIA results may lead to inaccurate conclusions on how diseases, habitat management, etc., affect the HPA-axis of bobwhite. Therefore, it is advised that future studies analysing FCM in wild bobwhite exclude faecal samples exposed to rainfall unless it is confirmed that rainfall does not affect bobwhite FCM levels. Further studies will need to be done to determine how faeces exposed to different environmental conditions affect the EIA outcomes.

In conclusion, we evaluated the impacts of three treatments of bobwhite faeces on the detected FCM levels. Our findings indicated that for results to be comparable using the EIA, the storage methods of faeces collected from male bobwhite must be fully standardized. We also found that FCM levels extracted immediately from faeces were not comparable to those from the other treatments, regardless of sex. This study enhances the applicability of the EIA for assessing differences in adrenocortical activity in bobwhite by identifying which faeces treatments yield comparable results.

## Supplementary Material

Web_Material_coaf051

## Data Availability

The data underlying this article will be shared on reasonable request to the corresponding author.
