# Supplementary material for: The effects of different sample storage conditions on faecal corticosterone metabolite measurements in northern bobwhite (Colinus virginianus)
Source: Conserv Physiol. 2025 Jul 29;13(1):coaf051. doi: 10.1093/conphys/coaf051 (PMC12305797; doi:10.1093/conphys/coaf051)
Supplement: Web_Material_coaf051 [file web_material_coaf051.zip › Supplemental_Info_1 (1).pdf]

Supplemental 1. The top six models from the model selection analysis with fixed parameters.

Fixed Model Parameters	Degrees of Freedom	Log Likelihood	AICc	ΔAIC	ω
*Sex + Treatment + Sex*Treatment*	9	-42.638	105.3	0	0.471
*Sex + Time + Treatment + Sex*Treatment + Sex*Time*	11	-40.622	106.3	1.05	0.279
*Sex + Time + Treatment + Sex*Treatment*	10	-42.563	107.6	2.32	0.147
*Sex + Time + Treatment + Sex*Treatment + Sex*Time + Time*Treatment*	13	-39.938	111.1	4.83	0.042
*Sex + Time + Treatment + Sex*Treatment + Time*Treatment*	12	-41.906	112.4	6.12	0.022
Treatment	6	-49.746	113	7.12	0.013
